# The Influence of Light Intensity and Leaf Movement on Photosynthesis Characteristics and Carbon Balance of Soybean

**DOI:** 10.3389/fpls.2018.01952

**Published:** 2019-01-08

**Authors:** Lingyang Feng, Muhammad Ali Raza, Zhongchuan Li, Yuankai Chen, Muhammad Hayder Bin Khalid, Junbo Du, Weiguo Liu, Xiaoling Wu, Chun Song, Liang Yu, Zhongwei Zhang, Shu Yuan, Wenyu Yang, Feng Yang

**Affiliations:** ^1^College of Agronomy, Sichuan Agricultural University, Chengdu, China; ^2^China Key Laboratory of Crop Ecophysiology and Farming System in Southwest, Ministry of Agriculture, Chengdu, China; ^3^Maize Research Institute, Sichuan Agricultural University, Chengdu, China; ^4^College of Resources, Sichuan Agricultural University, Chengdu, China

**Keywords:** light intensity, sucrose synthase, soybean, starch synthase, photosynthesis

## Abstract

In intercropping systems shading conditions significantly impair the seed yield and quality of soybean, and rarely someone investigated the minimum amount of light requirement for soybean growth and development. Therefore, it is an urgent need to determine the threshold light intensity to ensure sustainable soybean production under these systems. An integrated approach combining morphology, physiology, biochemistry and genetic analysis was undertaken to study the light intensity effects on soybean growth and development. A pot experiment was set up in a growth chamber under increasing light intensity treatments of 100 (L_100_), 200 (L_200_), 300 (L_300_), 400 (L_400_), and 500 (L_500_) μmol m^−2^ s^−1^. Compared with L_100_, plant height, hypocotyl length, and abaxial leaf petiole angle were decreased, biomass, root:shoot ratio, and stem diameter were increased, extremum was almost observed in L_400_ and L_500._ Leaf petiole movement and leaf hyponasty in each treatment has presented a tendency to decrease the leaf angle from L_500_ to L_100_. In addition, the cytochrome content (Chl a, Chl b, Car), net photosynthetic rate, chlorophyll fluorescence values of *F*_v_/*F*_m_, $F_{\text{v}}{}^{\prime }$/$F_{\text{m}}{}^{\prime }$, *ETR*, Φ_PSII_, and *qP* were increased as the light intensity increased, and higher values were noted under L_400_. Leaf microstructure and chloroplast ultrastructure positively improved with increasing light intensity, and leaf-thickness, palisade, and spongy tissues-thickness were increased by 105, 90, and 370%, under L_500_ than L_100_. Moreover, the cross-sectional area of chloroplast (C) outer membrane and starch grains (S), and sectional area ratio (S:C) was highest under L_400_ and L_500_, respectively. Compared to L_100_, the content of starch granules increased by 35.5, 122.0, 157.6, and 145.5%, respectively in L_400_. The same trends were observed in the enzyme activity of sucrose-synthase, sucrose phosphate synthase, starch synthase, rubisco, phosphoenol pyruvate carboxykinase, and phosphoenol pyruvate phosphatase. Furthermore, sucrose synthesis-related genes were also up-regulated by increasing light intensity, and the highest seed yield and yield related parameters were recorded in the L_400_. Overall, these results suggested that 400 and 500 μmol m^−2^ s^−1^ is the optimum light intensity which positively changed the leaf orientation and adjusts leaf angle to perpendicular to coming light, consequently, soybean plants grow well under prevailing conditions.

## Introduction

Light intensity and quality are among the most critical environmental factors for crop physiology and biochemistry (Yang et al., 2018a). For most crop plants, even a slight increase or decrease in light intensity leads to considerable changes in leaf morphology and structure (Wu et al., 2017). According to past comparative studies, the dry matter of roots, stems, leaves, and whole plant as well as the photosynthetic rate, transpiration, and stomatal conductance, and the stem diameter decreased in low light conditions (Yang et al., 2014, 2017). In addition, crop plants produce smaller and thinner leaves under low light conditions than corresponding leaves in full sunlight conditions (Wu et al., 2017). However, shading environments increased the plant height and lodging rate which hinders the transportation of nutrients, water, and photosynthetic products and ultimately causes huge losses to agriculture production. Taken together, light intensity is the main factors which controls the central process of plants such as germination, leaf proliferation and expansion, photosynthesis, buds and flower initiation, and cell division (Kong et al., 2016; Wu et al., 2018; Yang et al., 2018b). Indeed, the numerous plant processes improve with increasing light intensity up to a moderate level which bring dramatic developmental and physiological changes to occur, leading to the rapid increase of these processes (Yang et al., 2015; Wu et al., 2016).

Reductions in light intensity could affect carbon balance of crop plant because the carbohydrate demand increases while its production decreases: rates of physiological processes rise while the photosynthetic yield reduces (Lichtenthaler et al., 1981). Accordingly, tolerance to shade stress increased at high net photosynthetic rate in C_3_ plants (Su et al., 2014). Moreover, the pattern of carbohydrates into expensive processes, like the biosynthesis of defense proteins (notably light-harvesting chlorophyll protein) raises with increasing shade density (Yang et al., 2018b). In line with this, Rijkers et al. (2010) concluded that plant defense to shade stress is increased at optimum nitrogen supply due to the high light-saturated photosynthetic nitrogen-use. The net photosynthetic rate is the major driver of crop plant carbon balance, optimum and continuous availability of light should also be considered into account to study the plant responses to shade stress.

Leaf positioning directly determines the light interception, it has been considered that phototropism of leaf could be a part of plant responses to shade stress. For example, in different crop species, variations in leaf angle evade the heat stress when maximal light intensity is available (Fu and Ehleringer, 1989; King, 1997; Falster and Westoby, 2003; Vasseur et al., 2011). In crop plants, hyponasty (upward movement of leaf) is one of the first leaf morphological responses to changing light intensity (Pharis and King, 1985). Hyponastic response changes widely among different species and is related to different environmental conditions encountered in collection sites, indicating an adaptive role for this character (van Zanten et al., 2009b). Furthermore, Arabidopsis rosettes showed the hyponastic growth and reduced the leaf angle under high temperature caused by excessive light interception with higher rate of transpiration which could contribute to reducing leaf temperature by enhancing transpiration (Gray et al., 1998; Franklin, 2009). Usually, low light or shading conditions induced the hyponasty response in plants (Hangarter, 1997; Maliakal et al., 1999; Smith, 2000), which is controlled by phytochrome and cryptochrome pathways (Smalle et al., 1997; Vandenbussche et al., 2003; Kozuka et al., 2005; Millenaar et al., 2009). Therefore, hyponasty has been believed to be a typical morphological response of shade avoiding syndrome, which allowed the plants to capture more sun-light and increase carbon gain under the light competition conditions (Pierik et al., 2004; Mullen et al., 2006; van Zanten et al., 2010). Previously, many scientists have studied the effects of light intensity on leaf positioning (van Zanten et al., 2009a) and hypocotyl growth pattern (Wherley et al., 2005). Importantly, decreased light intensity induced the hyponastic response in multiple loss-of-function photoreceptor mutants (Millenaar et al., 2009). Taken together, these findings indicating a tight and close link between leaf angle and light intensity. Therefore, it is important to investigate the response of plant leaf angle under changing light intensity to understand the soybean leaf angle orientation under shading conditions. Crop growth as dry matter production is largely dependent on current photosynthesis and, therefore, one of the main important changes that shade stress affects crop growth is ascribed to its huge reduction of net photosynthesis (Yang et al., 2018b). Reductions in photosynthesis could occur by two main principle mechanisms (Yang et al., 2017): (i) decrease CO_2_ diffusion into leaves, due to the decrease internal and stomatal conductance (g_i_ and g_s_, respectively), and (ii) metabolic potential inhibition for photosynthesis by inhibiting the leaf growth and enlargement by controlling the cell proliferation (Wu et al., 2017, 2018). In addition, light is the only source of energy for starch biosynthesis (Stitt and Zeeman, 2012), and the rates of starch biosynthesis and degradation are adjusted to the availability of sunlight, so that when the availability of light increased the starch formation increased and the rate of degradation decreased (Fernandez et al., 2017). Actually, the starch content reduced in sugar beet (*Beta vulgaris*) and bean leaves as the intensity of light reduced (Fondy et al., 1989; Servaites et al., 1989). However, rarely researchers have investigated the effect of different light intensity on the biosynthesis of starch. Therefore, further investigations are needed due to the relative significance of such mechanisms is debatable.

The amount and activity of important enzymes involved in CO_2_ fixation and regeneration of rubisco-1, 5-bisphosphate (RuBP) determined the metabolic potential of photosynthesis in plants under different conditions (Seemann and Sharkey, 1986; Delfine et al., 1999; Redondo-Gomez et al., 2007) as well as the activity and content of light capturing components, electron transport fragments, and energy transferring enzymes (Kao et al., 2003; Ranjbarfordoei et al., 2006; Stepien and Kłbus, 2006). In photosynthesis Rubisco (RuBP carboxylase or oxygenase) catalyzes the process of CO_2_ fixation (Mauser et al., 2001), which is directly involved in first the phase of Calvin Benson cycle and accounting for 12–35% of the leaf protein especially in C_3_ crop plants (Evans and Seemann, 1989). In past reports, it have revealed that the main biochemical restraint involved in shade-associated down regulation of net photosynthetic rate was reduction in the amount or activity of Rubisco (Seemann and Sharkey, 1986).

In past few years, chlorophyll fluorescence measurements have been known as an informative and useful indicator characterizing different light responses of photosynthesis. Considerable attention was paid to investigate and to determine the important characteristics of this technique (Schreiber et al., 1995). Chlorophyll fluorescence mainly and effectively used to measures the quantum yield of photosystem II and photo-inhibition by determining the potential quantum yield under prevailing light and shade conditions (Rascher et al., 2010). Shade significantly affected the performance and structure of the photosynthetic apparatus (Yao et al., 2017). It blocks the energy transport from PSII to PSI, reduces the leaf thickness, palisade and spongy tissues which results in low chlorophyll fluorescence (Wu et al., 2017; Yao et al., 2017). Thus, in this present study we investigated the effects of different light intensity treatments on leaf positioning and internal structure, the changes in ultra-structure of chloroplast, photosynthetic and chlorophyll fluorescence parameters, leaf carbon status through sucrose and starch contents, enzymatic activity of key enzymes related to photosynthetic process and sugar synthesis, a transcriptional analysis of some targeted genes to investigate the minimum amount of light quantity required by soybean plants for optimum growth and development. Finally, we concluded that changes in yield and yield related components are tightly related to the changes in light quantity.

## Materials and Methods

### Plant Material and Growth Condition

*Glycine max* (Linn.) Merr. seeds (Nandou 12, a major soybean cultivar widely planted in Southwest China) were chosen for the phenotypic responses to growth conditions. Before the experiment, soybean seeds were cleaned by 75% ethanol and deionized water for 5 min and germinated on a wet sterile gauze for 48 h in the dark at 25°C. After germination, two seeds were sown in 400 mL pots filled with a mixed matrix of PINDSTRUP organic soil (Pindstrup Mosebrug A/S, Ryomgaard, Denmark) and vermiculite (v:v, 4:1) in a light chamber with 25°C/20°C day/night temperature, 60% relative humidity, 460 μmol mol^−1^ CO_2_ and 10/14 h photoperiod. In this experiment, we used growth chamber I-66VL (Percival Scientific Inc., Watson, American), three plates were placed in each chamber and every plate contained 5 plants at a distance of 20.4 cm, and for light intensity treatments we used LED lights. The soybean seedlings have grown up to V2 stage (before the second trifoliate leaf appearance) (Fehr and Caviness, 1977), the pots were transferred to 5 light chambers for light intensity treatment (Supporting Information S1, S2, and S3) by keeping the light quality same in all experimental treatments. When the four trifoliate leaf appearance (about 15 days), the second and third leaves will be harvest for parameters measurement and analyzed. The pots were moved daily to avoid boundary effects and soybean seedlings watered with a one-fifth-strength Hoagland's solution (Hoagland and Arnon, 1950) every 2 days. Light intensity and spectral irradiance (λ = 380–760 nm) measured by HR350 (Hipoint Inc., Gaoxiong, Taiwan). Every treatment was performed with three replicates.

### Plant and Leaf Traits

Total plant height, stem diameter, biomass, and root: shoot ratio of fully expanded leaf were determined by the light intensity conditions for 15 days treatment and measured at V4 stage. Measurement of abaxial leaf petiole angle (degree) were performed every 2 h in a day at on randomly selected plants.

### Photosynthesis and Photosynthetic Pigment Concentration

Li-6400 portable photosynthesis system (LI-COR Inc., Lincoln, NE, USA) was used for photosynthetic parameters measurement of soybean V3 expanding leaves. All parameters including net photosynthetic rate (*P*_n_), transpiration rate (*T*_r_), stomatal conductance (*G*_s_), and intercellular CO_2_ concentration (*C*_i_) were measured under steady light intensity 600 μmol m^−2^ s^−1^, environment temperature 25°C and a CO_2_ concentration 460 (μmol mol^−1^) from 9:00 to 11:00.

Soybean V3 trifoliate leaves were collected for photosynthetic pigment concentration measurement and each treatment in three replicates. Chl a, Chl b, Car were extracted from all the leaf samples, and two leaf discs (1.130 cm^2^) were cut from the middle part of each middle lobules by a puncher (1.2 diameter), and dipped the samples in 10 mL of N,N-dimethyl formamide solution in the dark for 48 h at 4°C (Kutík et al., 2001). The extraction mixture was then measured at wavelengths of 663, 645, and 470 nm by using a spectrophotometer DU-730 (Beck Man Coulter Inc., USA).

### Chlorophyll Fluorescence Measurements

The chlorophyll a fluorescence measurement was performed with the miniaturized pulse-amplitude-modulated photosynthesis yield. Before measurement, each plant were transferred to adapt for 30 min in the dark chamber and submitted to chlorophyll fluorescence image capture system (CFImager, Technologica Ltd., Colchester, UK) to estimate maximal PSII quantum yield (*F*_v_/*F*_m_), photochemical efficiency of PSII ($F_{\text{v}}{}^{\prime }$/$F_{\text{m}}{}^{\prime }$), effective PSII quantum yield (Φ_PSII_), non-photochemical quenching (*NPQ*) and coefficient of photochemical quenching (*qP*). All the parameters were calculated as the methods reported by Maxwell and Johnson (2000). The apparent electron transport rate (*ETR*) were defined by a uniform absorption of incident light over the whole middle trifoliate leaf at V3 stage.

### Leaf Anatomical Features and Chloroplast Ultrastructure

Leaf piece (4 mm^2^) about 15 replications for each treatment without midribs were fixed in formalin-acetic acid-alcohol solution (FAA; 90% ethanol; 5% formaldehyde; 5% glacial acetic acid, v/v/v) at 4°C for 3 days. The leaf samples were dehydrated in a graded series of ethanol solutions (95, 75, 50, 25, 10% of ethanol for each treatment 30 min and 3 times, respectively), embedded in paraffin and cut the tissue sections to 4 μm thickness with a rotary microtome (RM2235, Leica Microsystems Ltd., Germany). The tissue sections were co-stained by Safranine and Fast Green, observed with a light microscope (ECLIPSE Ts2, Nikon Instruments Inc., Japan) and captured by a digital camera (Digital Sight DS-U3, Nikon Instruments Inc., Japan). The thickness of total leaf, spongy, and palisade mesophyll, and abaxial epidermis were measured by ImageJ.A_S_ described by Yang et al. (2018b), same leaf piece size and replications were cut for chloroplast ultrastructure observation, fixed in glutaric dialdehyde solution (3% glutaraldehyde; 1% osmium tetroxide), and dehydrated in a graded series of acetone solutionsEpon812 were used for tissue embedment, then ultrathin section was cut after uranyl acetate and lead citrate staining. A transmission electron microscope (TEM; HITACHI, H-600IV, Japan) were used for photographs examination. The cross-sectional area of chloroplast, grana, thylakoids, and starch grains were measured by ImageJ.

### Real-Time Quantitative PCR Verification

The expanding leaves of Nandou12 at V4 stage under 5 light intensity conditions were harvested on five plants for consideration of RNA abundance and sensitivity of the blade to light intensity. All the leaves were labeled and frozen in liquid nitrogen immediately. RNA was extracted using the TRIzol™ Plus RNA Purification Kit (Invitrogen). Reverse transcription and amplification of cDNA were performed using Super Script III First-Strand Synthesis Super Mix for qRT-PCR (Invitrogen). Real-time quantitative PCR was conducted in Quant Studio 6 Flex Real-Time PCR System (Thermo Fisher Scientific) and 2^−ΔΔ*CT*^ method used for data analysis. Cycle threshold (C_t_) values were determined by subtracting the difference of the *Ct* levels. The reference gene *Gmactin11* was selected for the control. All the target genes primers are listed in Supporting Information Table S1.

### Enzyme Activity

Before the enzyme activity measurement, the frozen leaves were ground with a pre-cooled mortar and pestle in 1.5 mL ice-cold buffer containing 50 mM Hepes-KOH (pH 7.5), 2 mM EDTA, 10 mM MgCl_2_, 10 mM dithiothreitol (DTT), 1% (w/v) Triton X-100, 10% (w/v) glycerol, 1% (w/v) bovine serum albumin (BSA), 5% (w/v) polyvinylpolypyrrolidine (PVPP), and 1 mM phenylmethylsulfonyl fluoride (PMSF). The extract was centrifuged at 13,000 g at 4°C for 10 min, and the supernatant was used immediately for activity assay. SS, SPS were determined in 1 mL reaction mixture containing and 500 μL enzyme extract at 34°C for 1 h, added 30% KOH to mixture in a boiling water bath for 10 min to completely inactivate and cooled to room temperature, then applied 3.5 mL 0.15% anthrone-sulfuric acid solution in the last reaction mixture at 40°C for 20 min, the increase in A_620_ was monitored. Rubisco total activity was measured by injecting 100 μL of the supernatant into 400 μL of an assay mixture consisting of 50 mM Tris-HCl (pH 8.0), 5 mM DTT, 10 mM MgCl_2_, 0.1 mM EDTA, and 20 mM NaH_14_CO_3_ (2.0 GBq mmol^−1^) at 30°C. After a 5 min activation period, the reaction was initiated by adding RuBP to 0.5 mmol L^−1^ and terminating after 30 s with 100 μL of 6 mol L^−1^ HCl.PEPC and PEPP were extracted according to Yang et al. (2012) with some modifications. PEPC was assayed in 1 mL reaction mixture consisting of 50 mM Tris-HCl (pH 8.0), 5 mM MnCl_2_, 2 mM DTT, 10 mM NaHCO_3_, 0.2 mM NADH, 5 unit NAD-MDH and 160 μL enzyme extract. The reaction was initiated by adding 2.5 mM phosphoenolpyruvate (PEP). PEPP was determined in 1 mL reaction mixture containing 100 mM imidazole-HCl pH 7.0, 50 mM KCl, 10 mM MgCl_2_, 0.05% (w/v) BSA, 2 mM DTT, 150 μM NADH, 1 unit LDH, 2 mM ADP and 100 μL enzyme extract. The reaction was initiated with 2 mM PEP, the increase in A_412_ was monitored. In addition, the activities of adenosine diphosphate glucose pyro-phosphorylase (ADPGPPase), uridine diphosphate glucose pyro-phosphorylase (UDPGPPase), and soluble starch synthase (SSS) were measured by using the previously mentioned method (Doehlert et al., 1988; Liang et al., 1994).

### Soluble Sugar, Sucrose, and Starch Content

The samples of soybean leaves at V3 stage were harvest at the end of the day or night and analyzed by enzymatic assay as in Vasseur et al. (2011).

### Statistical Analysis of Data

All data analyses were performed by Statistical software (version 7.0; StatSoft). Significance was determined via one-way analysis of variance. Values are presented mean + standard error (SE) from three independent biological replicates, and the least significance difference (LSD) test was employed to compare the means at 5% probability level.

## Results

### Morphological Characteristics

**Figure 1:**
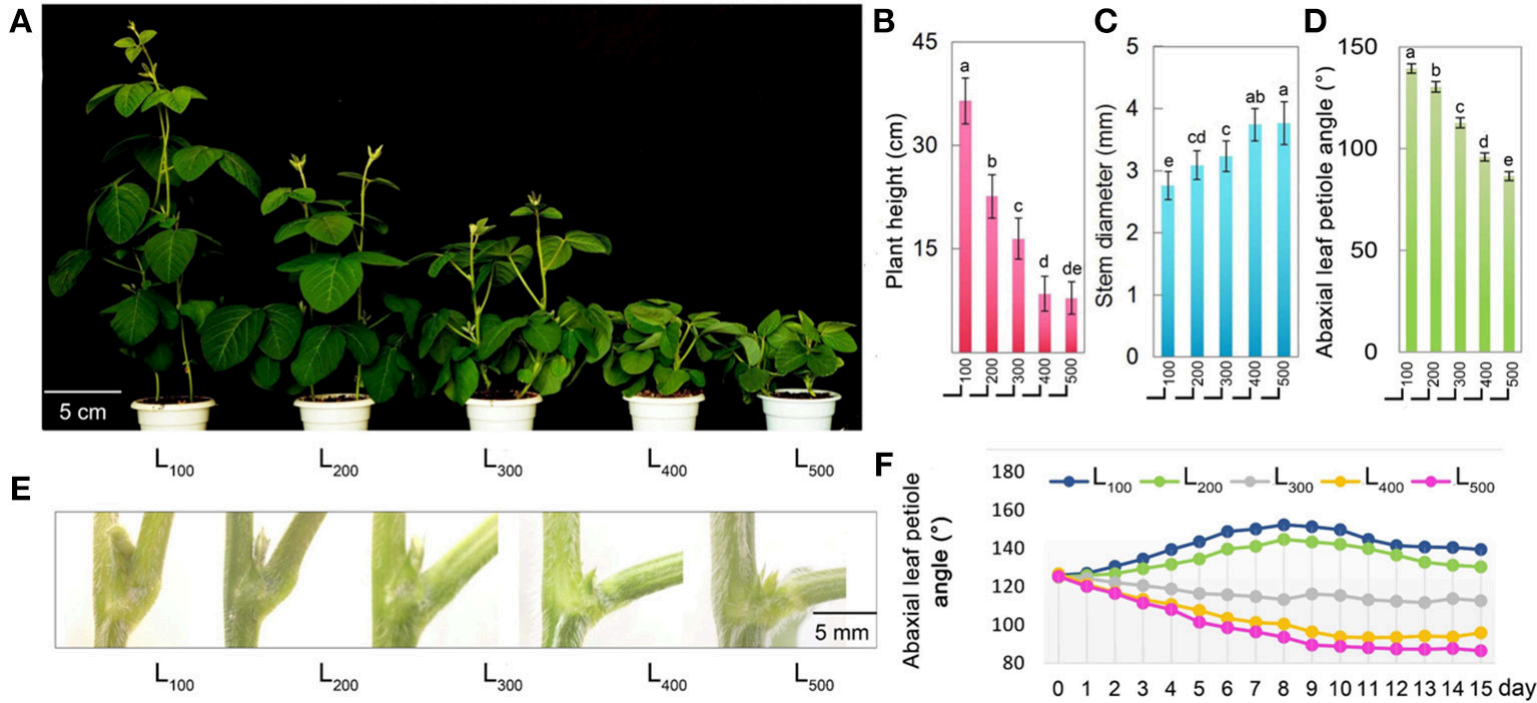
Changes in phenotype and plant traits of soybean as affected by different light intensity treatments. The phenotype **(A)**, Plant height **(B)**, stem diameter **(C)**, and abaxial leaf petiole angle **(D)**, leaf angle picture representation **(E)**, and graphical representation of leaf angle for continuous 15 days **(F)** of soybean plants under different light intensity treatments. L_100_, L_200_, L_300_, L_400_, and L_500_, refer 100, 200, 300, 400, and 500 μmol m^−2^ s^−1^, respectively. All the values are representative of three independent experiments. Bars show + standard errors. Within a bar, different lowercase letters show a significant difference at 5% level, between treatments.

Figure 1 presents the plant morphological characteristics of the soybean plants under different light intensity treatments. In the present experiment, different light intensity treatments had a significant effect on plant morphological characteristics, the soybean plant height and stem diameter of soybean were considerably increased from L_100_to L_500_. Specifically, the maximum soybean plant height 36.5 cm and stem diameter 3.86 mm were measured in treatments L_100_ and L_500_, and minimum plant height 7.9 cm and stem diameter 2.76 mm were observed under L_500_ and L_100_, respectively. Overall, treatment L_100_ and L_500_ significantly increased the plant height and stem diameter increased by 362 and 40%, with respect to L_500_ and L_100_, respectively. Table 1 presents the hypocotyl length, plant biomass (PB), and root to shoot ratio of soybean plants in response to different light intensity treatments. The light intensity treatments showed a significant effect on hypocotyl length, PB, and root to shoot ratio of soybean plants, and the PB, and root to shoot ratio of soybean plants in L_500_ were significantly higher than those under L_100_, whereas opposite trend was observed for hypocotyl length and it was maximum in L_100_ and minimum under treatment L_500_. Mostly, the plant traits showed were non-significant differences in treatments L_400_ and L_500_. These results present that increasing light intensity from L_100_ to L_500_ not only increased the stem diameter of soybean plants but also improves the other plant traits related to plant growth and development by reducing the plant height because higher plant height increases the rate of plant lodging under shading conditions. In addition, for plant morphological characteristics, vertical elongation of a plant was inhibited with the increase in light intensity, while the transverse extension was improved.

**Table 1:** Effect of different light intensity treatments on hypocotyl length (mm), biomass (g plant^−1^), and shoot to root ratio of soybean plants.

**Treatment**	**Hypocotyl length (mm)**	**Biomass (g plant^−1^)**	**Root/Shoot ratio**
L_100_	7.6 ± 0.70a	0.931 ± 0.14b	0.16 ± 0.02b
L_200_	6.0 ± 0.41b	1.228 ± 0.25ab	0.28 ± 0.10ab
L_300_	5.5 ± 0.35bc	1.048 ± 0.15ab	0.29 ± 0.03ab
L_400_	4.4 ± 0.35cd	1.441 ± 0.11a	0.38 ± 0.20a
L_500_	3.7 ± 0.39d	1.296 ± 0.19ab	0.36 ± 0.04ab

*L_100_, L_200_, L_300_, L_400_, and L_500_, refer 100, 200, 300, 400, and 500 μmol m^−2^ s^−1^, respectively. All the values are representative of three independent experiments. Bars show ± standard errors. Within a bar, different lowercase letters show a significant difference at 5% level, between treatments*.

### Leaf Anatomy

**Figure 2:**
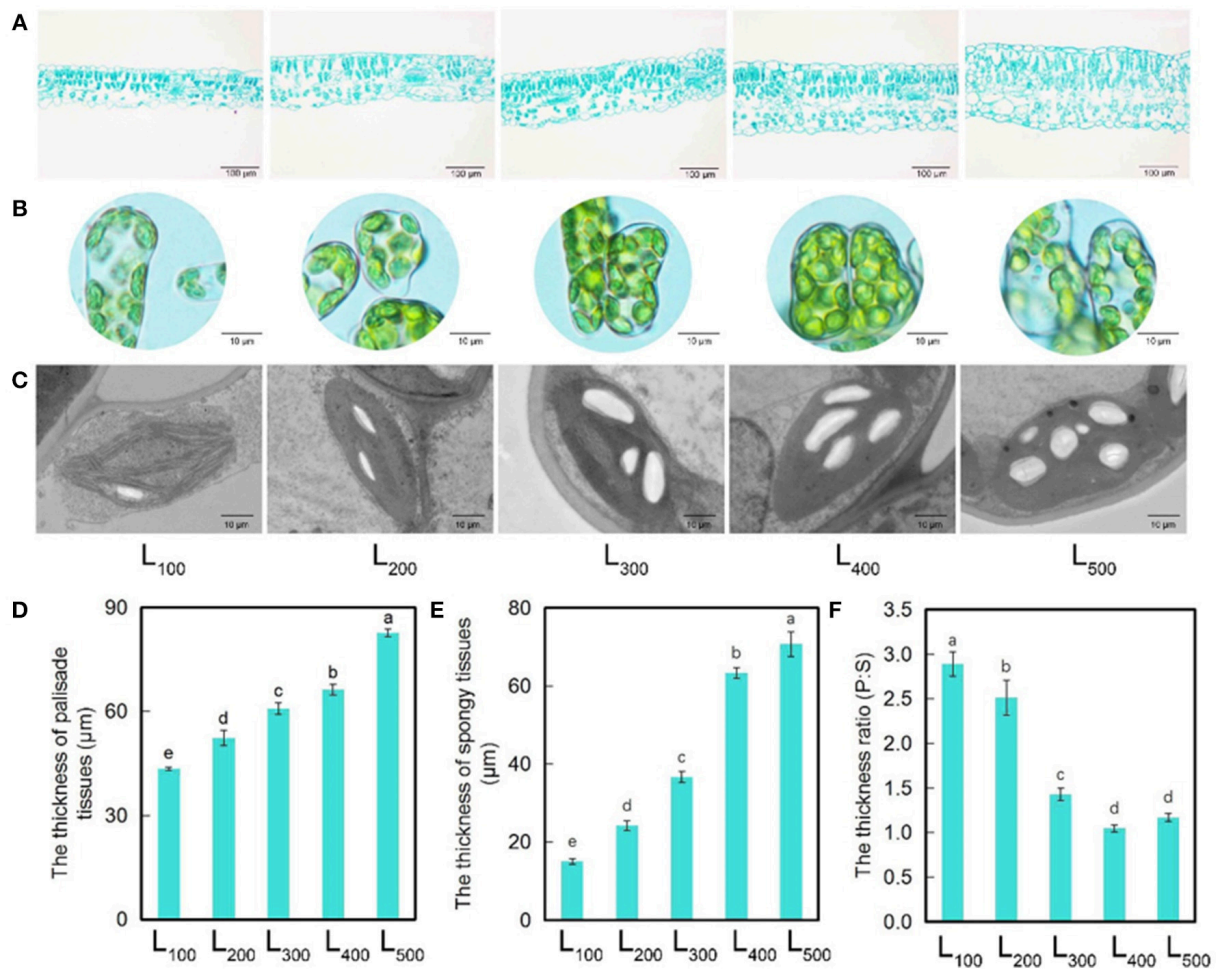
Changes in leaf structure and chloroplast structure of soybean plants as affected by different light intensity treatments. Leaf thickness **(A)**, chloroplast number **(B)**, starch grana **(C)**, thickness of palisade **(D)**, thickness of spongy tissues **(E)**, and ratio of palisade, and spongy thickness **(F)** of soybean plants under different light intensity treatments. L_100_, L_200_, L_300_, L_400_, and L_500_, refer 100, 200, 300, 400, and 500 μmol m^−2^ s^−1^, respectively. All the values are representative of three independent experiments. Bars show + standard errors. Within a bar, different lowercase letters show a significant difference at 5% level, between treatments.

Light quality and quantity, especially light intensity affect positively the leaf anatomy. In this study, the significant differences in leaf thickness, palisade tissues thickness, spongy tissues thickness, and ratio of palisade and spongy thickness were noticed among all the five light intensity treatments (Figures 2A,D–F). Interestingly, soybean leaves showed perfect development of the palisade tissues, and the clearer and compact structure of spongy tissues were observed under treatments L_400_ and L_500_. Moreover, the leaf thickness, palisade tissues thickness, and spongy tissues thickness of soybean plants under L_500_ were significantly higher than those in L_100_. The maximum and minimum leaf thickness, palisade tissues thickness, and spongy tissues thickness were noted under treatments L_100_ and L_500_, respectively. However, the soybean plants grown under L_400_ and L_500_ treatments exhibited the decreased ratio of palisade and spongy thickness than treatment L_100_. On average, leaf thickness, palisade tissues thickness, and spongy tissues thickness increased by 105, 90, and 370%, under L_500_ in comparison with L_100_ treatment, the ratio of palisade and spongy thickness was decreased by 147% in L_500_ than L_100_ treatment. These findings indicated that shade conditions or low light intensity negatively affected the soybean leaf tissue size, while an optimum light intensity significantly increased the leaf thickness, palisade tissues thickness, and spongy tissues thickness.

### Leaf Angle and Carbon Balance

Differences in light intensity induced the epinasty or hyponasty leaf movements in plants. In the present study, different light intensity treatments had a significant impact on the abaxial leaf petiole angle of soybean leaves (Figure 1D). A strong hyponasty (an increase in abaxial leaf petiole angle) was observed under light intensity treatments L_100_ and L_200_. The decrease in light intensity from L_500_ to L_100_ increase the hyponastic response in soybean plants, and minimum 87.5° abaxial leaf petiole angle was measured under L_500_ treatments, while maximum 141.6° abaxial leaf petiole angle was noted in L_100_ treatment. In addition, we measured abaxial leaf angle for continuous 15 days and all the treatments showed consistent effect on leaf angle of soybean plants, maximum under L_100_, and minimum in treatment L_500_ (Figures 1E,F). Overall, higher light intensity treatment decreased abaxial leaf petiole angle by 38% compared to the corresponding value under treatment L_100_. Our results indicated that the shading conditions increased the abaxial leaf petiole angle which negatively affected the light absorption and photosynthetic process in soybean plants.

**Figure 3:**
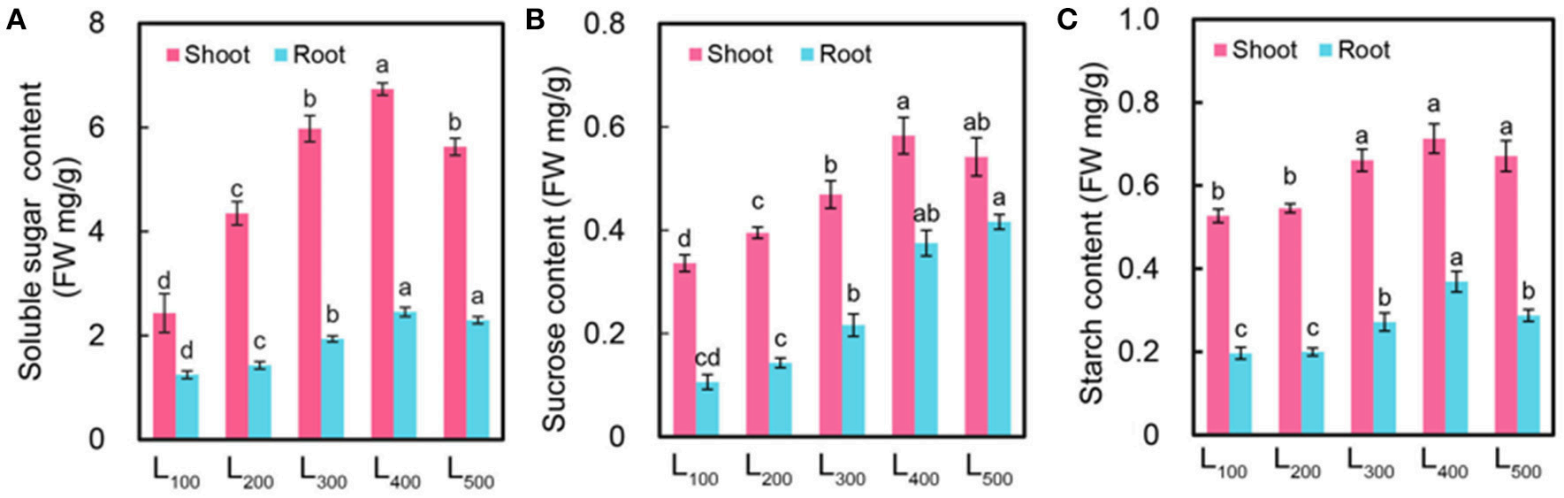
Changes in carbon balance of soybean plants as affected by different light intensity treatments. Soluble sugar content [FW (fresh weight) mg/g] **(A)**, sucrose content [FW (fresh weight) mg/g] **(B)**, and starch content [FW (fresh weight) mg/g] **(C)** of soybean plants under different light intensity treatments. L_100_, L_200_, L_300_, L_400_, and L_500_, refer 100, 200, 300, 400, and 500 μmol m^−2^ s^−1^, respectively. All the values are average of three replicates and representative of three independent experiments. Bars show + standard errors. Within a bar, different lowercase letters show a significant difference at 5% level, between treatments.

To further investigate the effect of light intensity solely regulates the hyponastic response in soybean, we determined the sucrose, starch, and total soluble sugar content of soybean shoot and root at the end of day and night. As expected, the sucrose, starch, and total soluble sugar content were significantly increased with increasing light intensity in both shoot and root. The highest sucrose content 0.58 mg g^−1^, starch content 0.71 mg g^−1^, and total soluble sugar content 6.74 mg g^−1^ were measured in treatment L_400_ as compared with L_100_ treatment in shoot (Figures 3A–C). The same trend was observed in root. Interestingly, under L_400_ treatment, this trend of increased sucrose, starch, and total soluble sugar content proved the epinastic movement of soybean leaves because as we mentioned above that increased light intensity (L_400_ and L_500_) decreased abaxial leaf petiole angle and increased the light absorption area of soybean leaves which in turn increased the sucrose, starch, and total soluble sugar content of shoot and root due to the higher photosynthetic activity in soybean plants.

### Chlorophyll Content and Chloroplast Structure

The chlorophyll (Chl) and carotenoids (Car) content of soybean leaves were considerably affected by different light intensity treatments. In this experiment, increasing light intensity from L_100_ to L_500_ increased the Chl a, Chl b, Chl a + b, and Car contents while Chl a/b decreased (Table 2). In addition, the Chl a, Chl b, Chl a + b, and Car contents of soybean leaves were found non-significant between light intensity treatments L_400_ and L_500_, but Chl a, Chl b, Chl a + b, and Car contents were always higher in L_500_ than those of under treatment L_100_. On average, Chl a, Chl b, Chl a + b, and Car contents increased by 43.6, 70, 49, and 20%, under L_500_ in comparison with L_100_ treatment, respectively. These improvements suggesting a direct relationship of chlorophyll and carotenoids content with the changes in light intensity.

**Table 2:** Effect of different light intensity treatments on cytochrome content (Chl a, Chl b, Car) and Chl a to b ratio of soybean plants.

**Treatment**	**Chl a (mg cm^**−2**^)**	**Chl b (mg cm^**−2**^)**	**Car (mg cm^**−2**^)**	**Chl a + b (mg cm^**−2**^)**	**Chl a/b**
L_100_	6.88 ± 0.11c	1.83 ± 0.03d	1.23 ± 0.04b	8.71 ± 0.16c	3.77 ± 0.03a
L_200_	8.17 ± 0.06b	2.53 ± 0.09c	1.38 ± 0.06ab	10.70 ± 0.06b	3.24 ± 0.14b
L_300_	8.22 ± 0.06b	2.69 ± 0.11bc	1.34 ± 0.02ab	10.91 ± 0.22b	3.06 ± 0.11b
L_400_	9.75 ± 0.14a	3.05 ± 0.05ab	1.45 ± 0.07a	12.80 ± 0.14a	3.19 ± 0.09b
L_500_	9.88 ± 0.12a	3.12 ± 0.11a	1.48 ± 0.04a	13.00 ± 0.28a	3.16 ± 0.07b

*L_100_, L_200_, L_300_, L_400_, and L_500_, refer 100, 200, 300, 400, and 500 μmol m^−2^ s^−1^, respectively. All the values are representative of three independent experiments. Bars show ± standard errors. Within a bar, different lowercase letters show a significant difference at 5% level, between treatments*.

In our study, the observations of chloroplast structure shown that chloroplast number and structure were significantly influenced by different light intensity treatments in soybean plants. The highest number of chloroplast was noticed under treatment L_400_, while the lowest number of chloroplast was observed under L_100_ (Figure 2B). Moreover, as compared to L_100_ chloroplasts under L_400_ were organized centrally in the cell and showed a more compact arrangement, and the grana stacks of chloroplast were clear and large, and every chloroplast contained 4–5 big starch grains (Figure 2C). In addition, the highest cross-sectional area of chloroplast (C) outer membrane, cross-sectional area of starch grains (S), thylakoid to chloroplast ratio (T:C), and sectional area ratio (C:S) were measured under L_500_ and L_400_, respectively, while the lowest was determined under treatment L_100_ Supporting Information Table S2. Taken together, increase light intensity significantly improved the chloroplast structure and arrangement.

### Enzymatic Activity and Gene Expression

**Figure 4:**
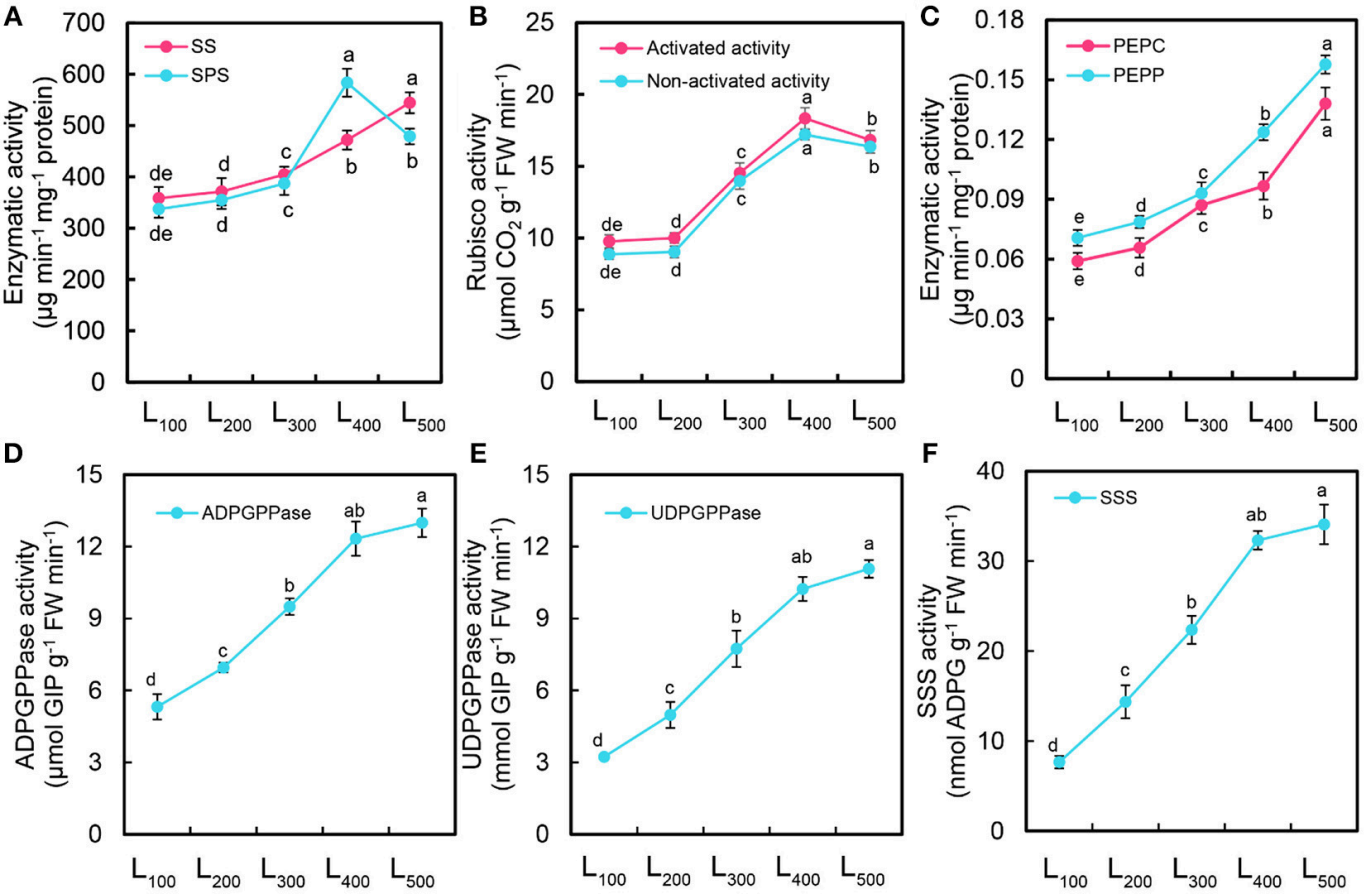
Changes in enzymatic activity of soybean plants as affected by different light intensity treatments. Activity of sucrose synthase (SS) and sucrose phosphate synthase (SPS) **(A)**, activated and non-activated activity of Rubisco **(B)**, activity of phosphoenolpyruvate carboxykinase (PEPC) and phosphoenolpyruvate phosphatase (PEPP) **(C)**, adenosine diphosphate glucose pyro-phosphorylase (ADPGPPase) **(D)**, uridine diphosphate glucose pyro-phosphorylase (UDPGPPase) **(E)**, and soluble starch synthase (SSS) **(F)** of soybean plants under different light intensity treatments. L_100_, L_200_, L_300_, L_400_, and L_500_, refer 100, 200, 300, 400, and 500 μmol m^−2^ s^−1^, respectively. All the values are representative of three independent experiments. Bars show ± standard errors. Within a bar, different lowercase letters show a significant difference at 5% level, between treatments.

In this experiment, significant differences in sucrose synthase (SS), sucrose phosphate synthase (SPS), rubisco, phosphoenol pyruvate carboxykinase (PEPC), phosphoenol lpyrute phosphatase (PEPP), adenosine diphosphate glucose pyro-phosphorylase (ADPGPPase), uridine diphosphate glucose pyro-phosphorylase (UDPGPPase), and soluble starch synthase (SSS) were noticed in different light intensity treatments (Figure 4). The SS, PEPC, PEPP, ADPGPPase, UDPGPPase, and SSS activities of soybean plants were gradually increased with increasing light intensity from L_100_ and L_500_ and the higher values were measured under L_500_ than those in other light treatments (Figures 4A–E). On average, the activities of SS, PEPC, PEPP, ADPGPPase, UDPGPPase and SSS were enhanced by 52, 134, 122, 144, 242, and 345% under L_500_, respectively, with respect to that in L_100_ treatment. Furthermore, we also measured the activities of SPS and rubisco, there was a significant difference among all light intensity treatments. Acceleration in the activities of SPS and rubisco occurred in all light treatments, the amplitude of acceleration was higher in L_400_ than L_500_, L_300_, L_200_, and L_100_ treatments. Moreover, we also measured the activities of starch biosynthesis enzymes (Supporting Information S4) and found maximum values from 400 to 500 μmol m^−2^ s^−1^. Overall, the SPS and rubisco activities of soybean plants under L_400_ was increased by 73 and 89% with respect to those under L_100_ treatment (Figures 4A,B). These results suggest that the activities of SS, PEPC, PEPP, SPS, and rubisco were directly associated with the changes in light intensity. In our case, using the L_400_ treatment can be more effective at the enzymatic activities.

**Figure 5:**
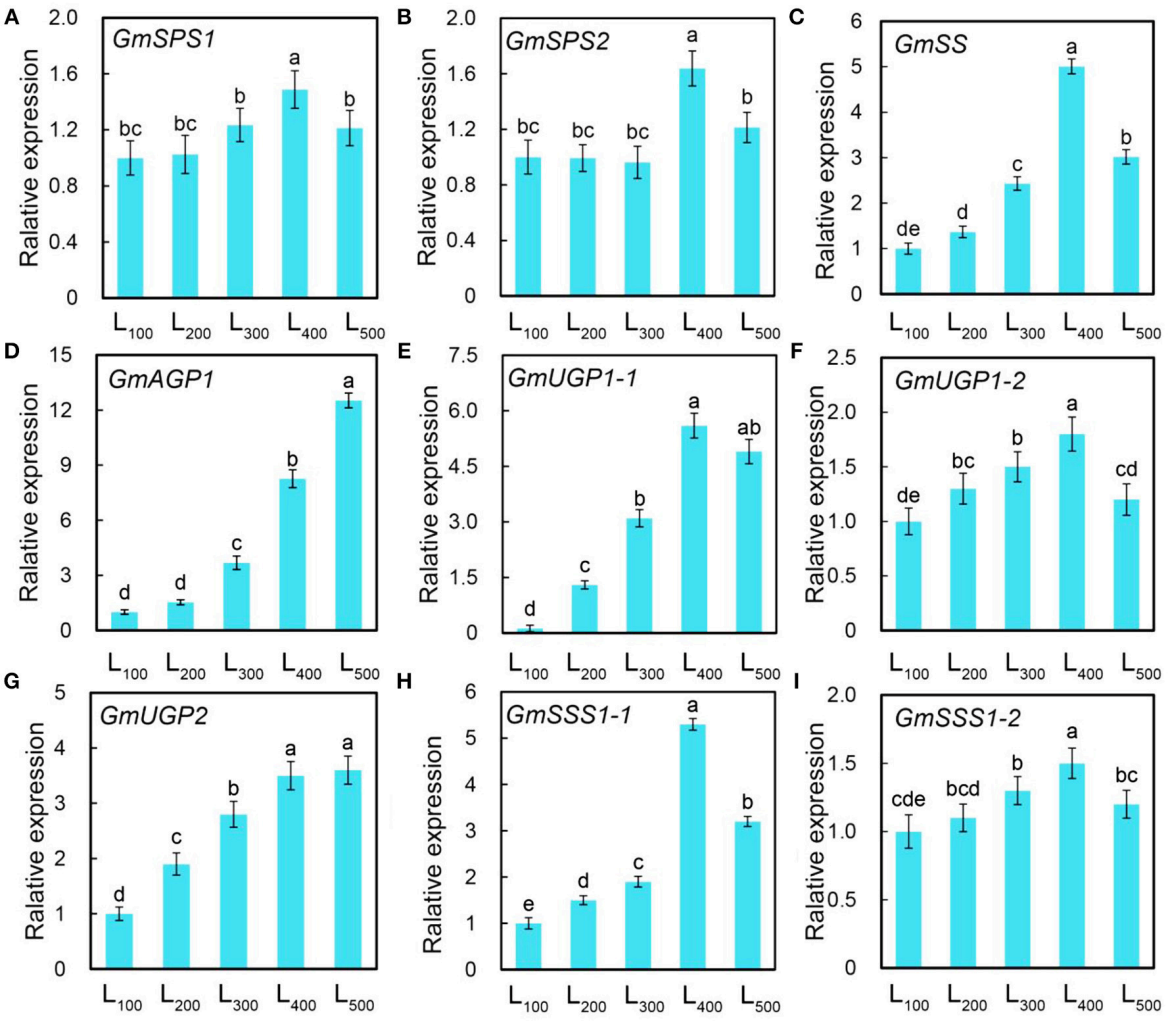
Changes in gene expression level of soybean plants as affected by different light intensity treatments. Gene expression of sucrose phosphate synthase (*GmSPSJ*) **(A)**, sucrose phosphate synthase (*GmSPS2*) **(B)**, sucrose synthase (*GmSSJ*) **(C)** starch synthase (*GmAGPJ*; **D**, *GmUGPJ-1*; **E**, *GmUGPJ-2*, **F**, *GmUGP2*,·**G**, *GmSSSJ-1*; **H**, and *GmSSSJ-2*,·**I**) of soybean plants under different light intensity treatments. L_100_, L_200_, L_300_, L_400_, and L_500_, refer 100, 200, 300, 400, and 500 μmol m^−2^ s^−1^, respectively. All the values are representative of three independent experiments. Bars show ± standard errors. Within a bar, different lowercase letters show a significant difference at 5% level, between treatments.

After blast against Arabidopsis, homologs of soybean were chosen to determine their gene expression levels in our experiment. Two genes expression involves in sucrose phosphate synthase (*Gmsps1* and *Gmsps2*), one gene involves in sucrose synthase (*Gmss1*), and six genes expression involves in starch synthase (*GmAGP1, GmUGP1-1, GmUGP1-2, GmUGP2, GmSSS1-1, and GmSSS1-2*) were quantitative analysis. The relative expression levels of all nine genes for sucrose phosphate synthase (2), sucrose synthase (1), and starch synthase (6) were up-regulated with increasing light intensity, and the expression of *GmSPS1* and *GmSPS2*, and *GmSS1*, and *GmAGP1, GmUGP1-1, GmUGP1-2, GmUGP2, GmSSS1-1*, and *GmSSS1-2*, were 1.5 and 1.6, and 5.0 (Figures 5A,B), and 8.3 (Figure 5C), 4.0, 1.8, 3.5, 5.3, and 1.5 (Figures 5D–I), respectively folds in treatment L_400_ compared with the L_100_ treatment.

### Photosynthetic and Chlorophyll Fluorescence Characteristics

Table 3 shows the photosynthetic (*P*_n_) characteristics of soybean plants in response to different light intensity treatments. The maximum *P*_n_, *G*_s_, and *C*_i_, and *T*_r_, values of soybean plants, appeared in treatments L_400_ and L_500_, respectively than those in L_100_, was 14.43 μ mol CO_2_ m^−2^ s^−1^, 0.59 mol H_2_O m^−2^ s^−1^, and 467.3 mol CO_2_ mol^−1^, and 1.93 mmol H_2_O m^−2^ s^−1^. *P*_n_, *G*_s_, and *C*_i_, and *T*_r_, of soybean plants, were increased by 58, 84, and 12%, and 57% under L_400_ and L_500_, respectively compared to the corresponding values under L_100_. This increase in net photosynthetic rate indicating that light intensity is positively related with the decrease abaxial leaf petiole angle and chlorophyll contents, as light intensity (L_400_ and L_500_) significantly decreased abaxial leaf petiole angle and chlorophyll contents in soybean plants.

**Table 3:** Effect of different light intensity treatments on photosynthetic characteristics of soybean plants.

**Treatment**	***P*_n_ (μmol CO_2_ m ^−2^ s^−1^)**	***Gs* (mol H_2_O m ^−2^ s^−1^)**	***Ci* (mol CO_2_ mol^−1^)**	***T*_r_ (mmol H_2_O m ^−2^ s^−1^)**
L_100_	9.13 ± 0.06d	0.32 ± 0.01d	418.93 ± 0.19c	1.23 ± 0.01d
L_200_	11.70 ± 0.06c	0.44 ± 0.01b	373.26 ± 0.30d	0.98 ± 0.01*e*
L_300_	13.47 ± 0.16b	0.37 ± 0.01c	415.14 ± 0.48c	1.30 ± 0.02c
L_400_	14.43 ± 0.01a	0.59 ± 0.02a	467.30 ± 0.05a	1.74 ± 0.01b
L_500_	14.41 ± 0.02a	0.44 ± 0.02b	436.67 ± 0.28b	1.93 ± 0.01a

*L_100_, L_200_, L_300_, L_400_, and L_500_, refer 100, 200, 300, 400, and 500 μmol m^−2^ s^−1^, respectively. All the values are representative of three independent experiments. Bars show ± standard errors. Within a bar, different lowercase letters show a significant difference at 5% level, between treatments*.

The fate of absorbed radiation energy in soybean leaves was studied in response to different light treatments (Table 4). In this experiment, the chlorophyll fluorescence parameters including *F*_v_/*F*_m_, *NPQ, qP*, Φ_PSII_, and *ETR* were significantly changed in different light intensity treatments. The *F*_v_/*F*_m_, *NPQ, qP*, Φ_PSII_, and *ETR* of soybean leaves under L_400_ and L_500_ were significantly higher than those in L_100_. Furthermore, L_400_ considerably increased the quantum yields of *F*_v_/*F*_m_, *NPQ, qP*, Φ_PSII_, and *ETR* by 9, 4, 24%, and 24, and 24% respectively, as compared to those under L_100_, indicating that improve light environment play an important role in improving the chlorophyll fluorescence parameters and photosynthetic capacity of soybean plants.

**Table 4:** Effect of different light intensity treatments on chlorophyll fluorescence characteristics of soybean plants.

**Treatment**	***F*_**v**_*/F*_**m**_**	***F*_**v**_'*/F*_**m**_'**	**NPQ**	**ETR**	**Φ_**PSII**_**	***qP***
L_100_	0.75 ± 0.009c	0.54 ± 0.002b	2.33 ± 0.02c	87.78 ± 6.2c	0.21 ± 0.002c	0.37 ± 0.01c
L_200_	0.76 ± 0.006c	0.56 ± 0.003b	2.37 ± 0.03c	92.82 ± 5.8c	0.22 ± 0.001c	0.38 ± 0.01c
L_300_	0.81 ± 0.005a	0.59 ± 0.004a	2.46 ± 0.05b	105.1 ± 6.9a	0.23 ± 0.002b	0.42 ± 0.01b
L_400_	0.82 ± 0.008a	0.58 ± 0.004a	2.43 ± 0.04b	109.2 ± 5.5a	0.26 ± 0.003a	0.46 ± 0.02a
L_500_	0.79 ± 0.009b	0.53 ± 0.002b	2.83 ± 0.06a	99.06 ± 5.1b	0.24 ± 0.001b	0.41 ± 0.01b

*L_100_, L_200_, L_300_, L_400_, and L_500_, refer 100, 200, 300, 400, and 500 μmol m^−2^ s^−1^, respectively. All the values are representative of three independent experiments. Bars show ± standard errors. Within a bar, different lowercase letters show a significant difference at 5% level, between treatments*.

### Yield and Yield Components

In our study, there was a significant impact of different light intensity treatments on seed yield of soybean plants Table 5. The highest seed yield, 10.1 g p^−1^, was recorded in the L_400_ treatment. Relative to L_100_, soybean plants in L400 obtained a higher seed yield. Yield components also changed among different treatments. The effects of light intensity on pod number per plant (PNP), seed number per plant (SNP) and 100-seed weight (SW) were significant, PNP and SNP under L_400_ were significantly higher than that in other treatments. Meanwhile, SW was considerably heavier in L_100_ as compared to L_400_ treatment. Overall, light treatment L_400_ increased the PNP and SNP by 70 and 64% as compared to L_100_, and L_100_ enhanced the SW by 19% than L_400_ treatment Table 5.

**Table 5:** Effect of different light intensity treatments on pod number per plant (PNP), seed number per plant (SNP), 100-seed weight (SW), and seed yield per plant (SY) of soybean plants.

**Treatment**	**PNP**	**SNP**	**100-SW (g)**	**SY (g plant^**−1**^)**
L_100_	23.0 ± 2.0b	30.3 ± 1.5d	25.3 ± 0.6a	7.7 ± 0.4cd
L_200_	24.3 ± 1.2b	35.7 ± 0.6c	23.7 ± 0.2a	8.4 ± 0.2bc
L_300_	34.3 ± 2.1a	47.0 ± 2.0b	20.5 ± 0.5bc	9.6 ± 0.3ab
L_400_	39.0 ± 2.0a	49.7 ± 2.1a	20.4 ± 0.1c	10.1 ± 0.4a
L_500_	25.0 ± 1.5b	32.7 ± 2.0d	23.3 ± 0.2ab	7.4 ± 0.3d

*L_100_, L_200_, L_300_, L_400_, and L_500_, refer 100, 200, 300, 400, and 500 μmol m^−2^ s^−1^, respectively. All the values are representative of three independent experiments. Bars show ± standard errors. Within a bar, different lowercase letters show a significant difference at 5% level, between treatments*.

## Discussion

### Variations in Light Intensity: Their Effects on Morphological Characteristics of Soybean

The morphology of crops has certain plasticity, and corresponding adaptation mechanisms exist under different environmental conditions (Gong et al., 2015). Use of higher plant population and intercropping systems are the effective ways for increasing the crop yields especially in developing countries (Xie et al., 2017). However, these methods are typically obstructed by reducing light conditions (Li R. et al., 2014). Numerous reports have confirmed that shade conditions promote the upward growth of stems and petiole while reducing the plant leaf area (Kurepin et al., 2007; Gommers et al., 2013). However, a few experiments pay attention to the impact of changing light intensity on plant morphology. In our experiment, a gradual increase in light intensity significantly improved the stem diameter, PB, and root to shoot ratio, and decreased the plant height and hypocotyl length of soybean plants. These results indicated that any change in light intensity directly affects the morphological parameters of soybean and low light conditions negatively affected the soybean morphology by increasing plant height and reducing stem diameter which in turn caused soybean lodging especially under intercropping conditions (Liu et al., 2016). Similarly, in previous study it has been reported that decrease light intensity significantly changed the soybean morphology by reducing plant dry matter production (Yang et al., 2014). In addition, the plant height, stem diameter, and PB of soybean showed varying responses to different light intensity treatments, and these parameters may be regulated by molecular regulation networks and endogenous plant hormones (Vandenbussche et al., 2005; Sheerin and Hiltbrunner, 2017). Overall, these results showed that L_400_ greatly improved the soybean morphology, and it is important to increase PB and stem diameter as compared to L_100_.

### Variations in Light Intensity: Their Effects on Leaf Anatomy of Soybean

Leaves are the main part of photosynthesis and any changes in leaf anatomy positively or negatively affected the plants photosynthesis under prevailing conditions. It is apparent that the environmental impacts on plant leaf structure changes for every environmental factor. Results of this study confirm earlier findings (Wu et al., 2017) that improve leaf structure can obtain in crop plants which have been planted under strong light than shading conditions. In addition, higher light intensity mostly increases leaf thickness, palisade tissues thickness, and spongy tissues thickness of leaves as seen in our experiment and previous studies (Fan et al., 2018). This improvement in leaf thickness maybe linked with the increase in mesophyll tissue and lower light intensity produced leaves with large cell gap and loose cell arrangement, therefore palisade tissues and spongy tissues thickness decreases, it might be due to the reduced cell growth and cell layer number in palisade tissues (Kalve et al., 2014). Moreover, the improve light intensity increased the palisade tissue elongation process which enhanced the chloroplast channel area through which carbon dioxide enters, consequently the thickness of leaves and photosynthetic capacity of soybean leaves significantly strengthen (Terashima et al., 2006; Wu et al., 2016) under L_400_ and L_500_ treatments. On average, the differences in leaf anatomy under different light intensity treatments suggesting that leaf structural components are the main targets of light and by making adjustments in leaf anatomy plants can perform better under shade stress conditions.

### Variations in Light Intensity: Their Effects on Leaf Angle and Carbohydrate Contents

**Figure 6:**
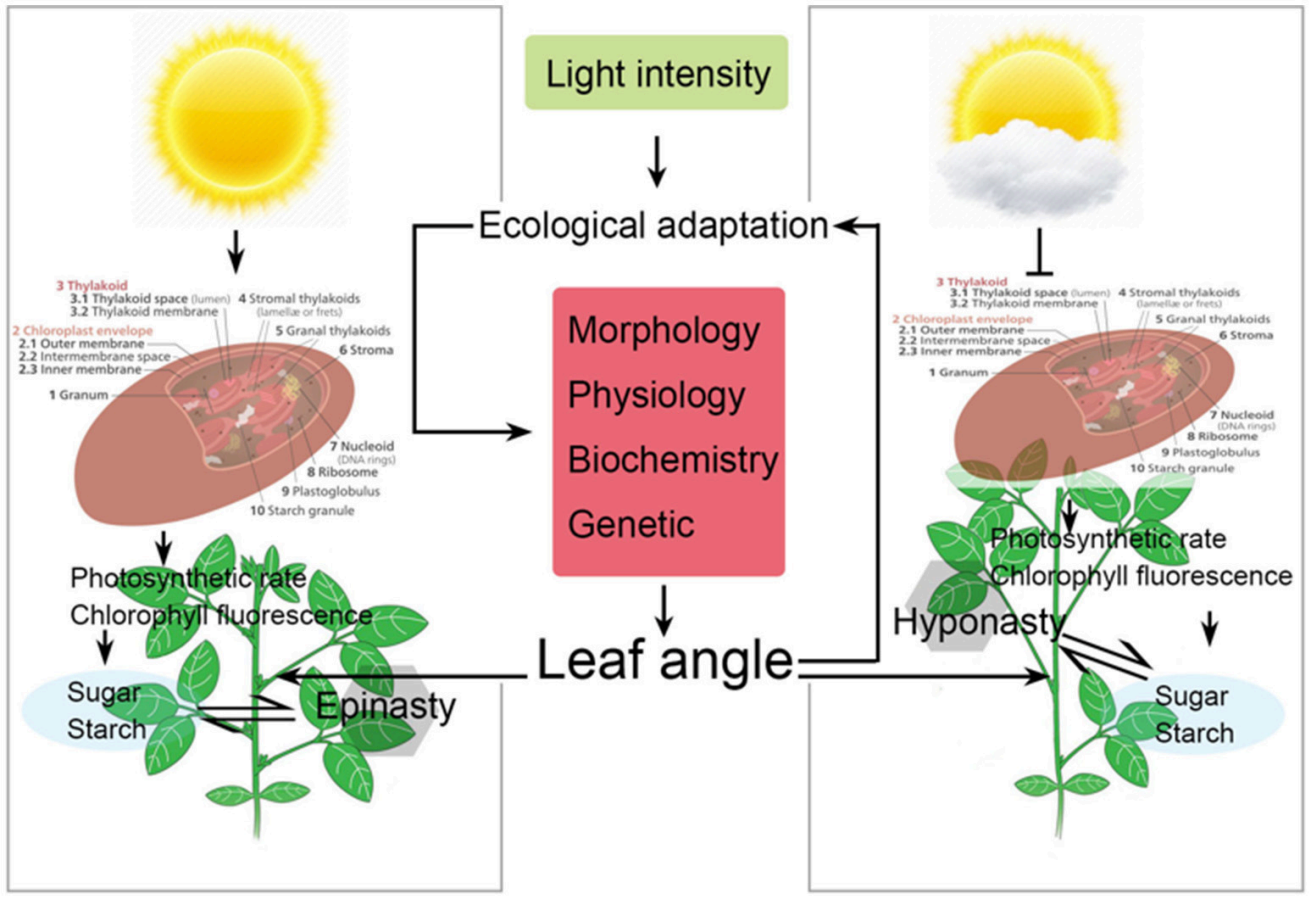
Schematic representation of changes in leaf orientation (leaf angle), physiology, and photosynthetic characteristics of soybean plants as affected by different light intensity treatments. Arrows represent the regulating directions of light intensity on soybean growth and carbon synthesis in this paper. Suppression arrow represent that reduce light intensity which negatively affect the differential growth and carbon synthesis in soybean plants by reducing the chloroplast efficiency.

The results of this experiment contribute to better understand soybean leaf angle and leaf movement (epinastic or hyponastic) behavior under changing light intensity treatments. Normally, leaf angle is neglected in studies of photosynthesis while it is the most important factor which influences the process of photosynthesis (Larbi et al., 2015). Obviously, crop leaves with orientations more horizontally to stem or perpendicular to solar radiations will absorb higher amount of solar radiations than those with more perpendicular to stem or parallel orientations to solar radiation (Lovelock et al., 1992); therefore, changes leaf angle can be indicator of plant response to different light intensities available under field conditions. In the present study, the higher light intensity significantly increased leaf angle by decreasing abaxial leaf petiole angle between stem (Figure 6) and leaf in L_500_ treatment than those of under L_100_. The increase in light intensity increased the PB as well as leaf biomass and area, which in result increase leaf angle due to the higher gravitational force on those leaves which had more leaf biomass as compared to those which had less leaf biomass. Similarly, Msallem (2002) reported that higher leaf angle under normal light than those under shading conditions. On the contrary, Larbi et al. (2015) found higher leaf angle under low photosynthetically active radiations (PAR) interception as compared with their corresponding values in higher PAR interception condition. However, in accordance to our results van Zanten et al. (2009c) confirmed that increasing light intensity decreased the abaxial leaf petiole angle and also restores many other plant traits associated with leaf structure (Millenaar et al., 2009; van Zanten et al., 2009c). Furthermore, carbohydrate content is the direct expression of the strong photosynthesis. Plants translocate sugar from photosynthesizing leaves to food storing cells that decides the physical fitness of plants (Amiard et al., 2005). Several past experiments concluded the role of light intensity for the synthesis of sucrose, starch, and total soluble sugar content in plants (Preiss, 1998; Michalska et al., 2009). In our results, the sucrose, starch, and total soluble sugar content were significantly increased with increasing light intensity. These results were in agreement with the previous studies (Pilkington et al., 2015). The increase in light intensity played an important role in regulating the enzymes related to sucrose and starch (Eliyahu et al., 2015). Similarly, former studies have reported that cloudy days and low light conditions reduced the soluble sugar content in leaves (Lichtenthaler et al., 1981; Mengin et al., 2017).

### Variations in Light Intensity: Their Effects on Chlorophyll Content and Chloroplast Structure

One of the plant leaf traits which is most affected by variations in light intensity or shading is Chl a, Chl b, Chl a + b, and Car contents. In our study, significant changes were observed in Chl a, Chl b, Chl a + b, and Car contents, and chlorophyll contents were increased with the increase in light intensity and these results were directly associated with leaf thickness (Figure 6), our results are consistent with previously reported results (Wittmann et al., 2001; Fan et al., 2018). Conversely, several other studies claimed that chlorophyll contents increase with the reduction in light intensity, especially contents of Chl b (Li T. et al., 2014).

Chloroplast ultrastructure controls the photosynthetic performance of crops under changing environmental conditions (Shao et al., 2014). In our study, the number of chloroplast and grana were increased significantly under higher light intensity L_400_ and L_500_ treatments as compared to lower light treatment L_100_, which suggests the beneficial effect of L_400_ and L_500_ treatments on photosynthetic apparatus of plant (chloroplast ultrastructure), our findings in line with the results of Yin et al. (2012). Anderson et al. (2008) reported the contradictory results and lower grana number in chloroplast was found under higher light intensity. Furthermore, the improved structure of chloroplast under higher light intensity treatments suggested that it might develop the shade-tolerant mechanism in soybean plants, especially under low light conditions. Therefore, the optimum light intensity (L_400_) improved the chloroplast ultrastructure and arrangement of soybean leaves.

### Variations in Light Intensity: Their Effects on Photosynthetic and Chlorophyll Fluorescence Characteristics

In addition to the effects of light intensity on morphology, leaf anatomy and chloroplast structure, our findings demonstrate that deleterious impacts of low light abolished by optimum light conditions. There are many reasons why crop plants in shading conditions carbon would be limited. For example, Yang et al. (2017) reported that soybeans assimilate demand increased while photosynthetic capacity decreased under shading conditions (Su et al., 2014). In this study, the increase in light intensity led to enhance the net photosynthetic rate, stomatal conductance, intercellular carbon dioxide levels, and transpiration rate of soybean plants. Thus, this showed that the improved photosynthetic parameters enhanced the carbon gain and promoted the soybean growth (Liao et al., 2005). Moreover, these results suggesting that the increase in net photosynthetic rate under L_400_ and L_500_ treatments may be due to the increase in stomatal opening and the changes in net photosynthetic rate were closely associated with the stomatal opening.

Increased photosynthetic capacity is always accompanied with high quantity of electrons passing through PSII (Yao et al., 2017). Chl fluorescence characteristics are one of the main important factors in photosynthetic regulation and plant responses to environmental conditions because of its sensitivity and convenience (Dai et al., 2009). Former studies have reported that low light intensity or shade results in low photosynthesis due to the reduction in qp, PSII and ETR while it increases the NPQ (Huang et al., 2011; Yao et al., 2017). In our present study, similar results were obtained, however, improved Chl fluorescence characteristics were measured under L_400_ and L_500_ treatments. These results reveal that higher light intensity enhances the efficiency of PSII and ETR that could enhance the photosynthesis by improving the energy transport from PSII to PSI (Figure 6), our results are consistent with previously reported results of Yang et al. (2018b).

### Variations in Light Intensity: Their Effects on Enzymatic Activity and Genes Expression

Results showed from this experiment demonstrating that the enzymatic activities of key enzyme related to sucrose synthesis (SS, SPS, and PEPC), starch synthesis (ADPGPPase, UDPGPPase, and SSS) and photosynthesis (rubisco) process significantly increased with increasing light intensity treatments, higher levels activities of these enzymes were determined under L_400_ and L_500_ treatments. These results are in line with the findings of previous reports (Bahaji et al., 2015). In addition, changes in light intensity equally played major roles in accelerating the activities of SS, SPS, and PEPC (Ciereszko et al., 2001). Therefore, plant biomass and net photosynthetic rate, which were largely regulated by improving light intensity might be affected the activities of SS, SPS, and PEPC, and ADPGPPase, UDPGPPase, and SSS, and controlled cell elongation and division in plants by regulating the expression of many genes. These results indicating that the higher sucrose and starch contents were increased by the activities of SS, SPS, and PEPC, and ADPGPPase, UDPGPPase, and SSS with other plant response to higher light intensity treatments, and in our case, using the L_400_ treatment, can be considered to more effective at the enzymatic activities. Furthermore, the loss of Rubisco activity was recognized to be very early and fast response of crop plants to shade stress (Servaites et al., 1986). Whereas, in this research the activity of Rubisco was increased with increasing light intensity, similar results were reported by Carmo-Silva and Salvucci (2013). This higher rubisco activity under higher light intensity treatments showed that the higher net photosynthetic rate of soybean plants directly correlated with rubisco activity under changing environments (Zhang et al., 2002). The relative expression levels of Gm*SPS1, GmSPS2, GmSS1, GmAGP1, GmUGP1-1, GmUGP1-2, GmUGP2, GmSSS1-1*, and *GmSSS1-2* were enhanced and increased the production of sucrose and starch to improve the soybean growth and development. The activities of SPS and SS, and ADPGPPase, UDPGPPase, and SSS enzymes were directly related with the upregulation of these important genes, therefore, in soybeans Gm*SPS1, GmSPS2, GmSS1, GmAGP1, GmUGP1-1, GmUGP1-2, GmUGP2, GmSSS1-1*, and *GmSSS1-2* were the important regulators of carbon production and of soybean better growth under low light conditions.

### Variations in Light Intensity: Their Effects on Yield and Yield Components

Previously, it has been reported that decrease light intensity or shading conditions significantly decreased the soybean yield and yield related parameters (Wu et al., 2016). However, in our study, increasing light intensity treatments had significant on yield and yield related parameters, and maximum PNP, SNP, and seed yield of soybean plants were recorded under L_400_ treatment as compared to other treatments. This is might be due to the higher net photosynthetic rate and biomass accumulation, similar to our results Yang et al. (2017) reported that soybean seed yield significantly reduced under shading conditions as compared to normal conditions. Moreover, light enrichment treatments significantly increased the pod number and seed yield of soybean reported by Liu et al. (2008). Hence, PNP and SNP of soybean plants might be improved under high light intensity treatments (L_400_ and L_500_), these results implied that higher light intensity at soybean canopy can significantly improve the morphological traits, leaf anatomical characteristics, photosynthetic, and chlorophyll florescence parameters which in turn considerably increased the seed yield by increasing the PNP and SNP.

## Conclusion

The significant effects of increasing light intensity on soybean plants have been extensively investigated, but rarely scientist have studied the impacts of different light intensity treatments on soybean to understand the optimum requirement of light for better growth and development. Here, we demonstrated that 400 μmol m^−2^ s^−1^ is the threshold level of light intensity which strongly interacts with soybean plant responses to treatment L_400_ by modifying its carbon balance (Figure 6). Increased light intensity significantly improved the morphological parameters, carbon assimilation rate (production of sucrose and starch), enzymatic activities of key enzymes by up-regulating the important sucrose synthase genes. These energetically expensive pathways positively modify carbon balance which is considerably improved with optimum light intensity. Because the dose response to changed light intensity varies between varieties and crop species, it is likely to play an important role in crop plant strategies and community dynamics. In addition, as compared to low light treatment (L_100_), higher light improved the leaf structure and anatomy which in turn significantly increased the photosynthetic and chlorophyll florescence especially quantum yield of PSII which in turn substantially increased the yield and yield related parameters. Therefore, we found that 400 and 500 μmol m^−2^ s^−1^ is the optimum light intensity which changed the leaf orientation and adjusts leaf angle to perpendicular to coming light, consequently soybean plants grows well under prevailing conditions.

## Author Contributions

LF, MR, and ZL performed the experiment. YC and MK performed some analysis. LF, MR, JD, WL, XW, CS, LY, ZZ, and SY conceived the original research plans. FY and WY designed the experiments, analyzed the data, and wrote the article. All authors read and approved the final manuscript.

### Conflict of Interest Statement

The authors declare that the research was conducted in the absence of any commercial or financial relationships that could be construed as a potential conflict of interest.
