# Supplementary material for: The Influence of Light Intensity and Leaf Movement on Photosynthesis Characteristics and Carbon Balance of Soybean
Source: Front Plant Sci. 2019 Jan 8;9:1952. doi: 10.3389/fpls.2018.01952 (PMC6338029; doi:10.3389/fpls.2018.01952)
Supplement: Supplementary file 1 [file Data_Sheet_1.docx]

**Supporting Information**

**Supplemental Figure**


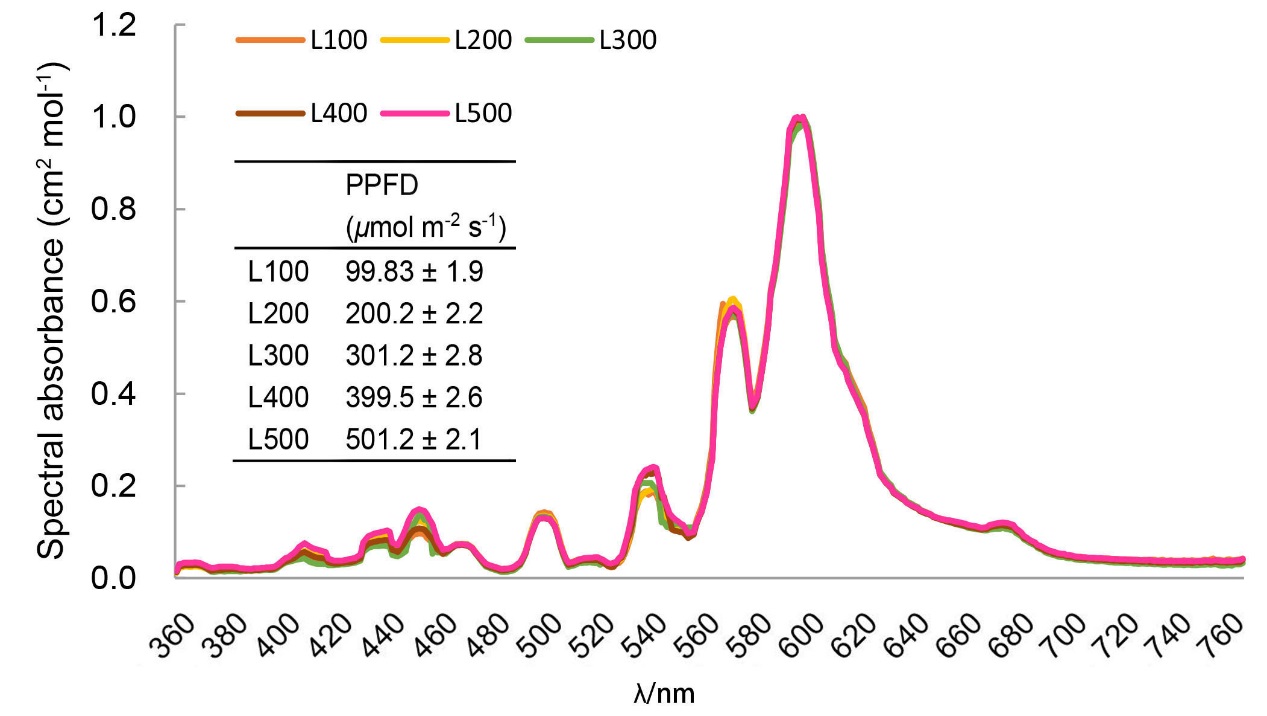


**S1 | Light quality of soybean canopy measured under different light treatments.**


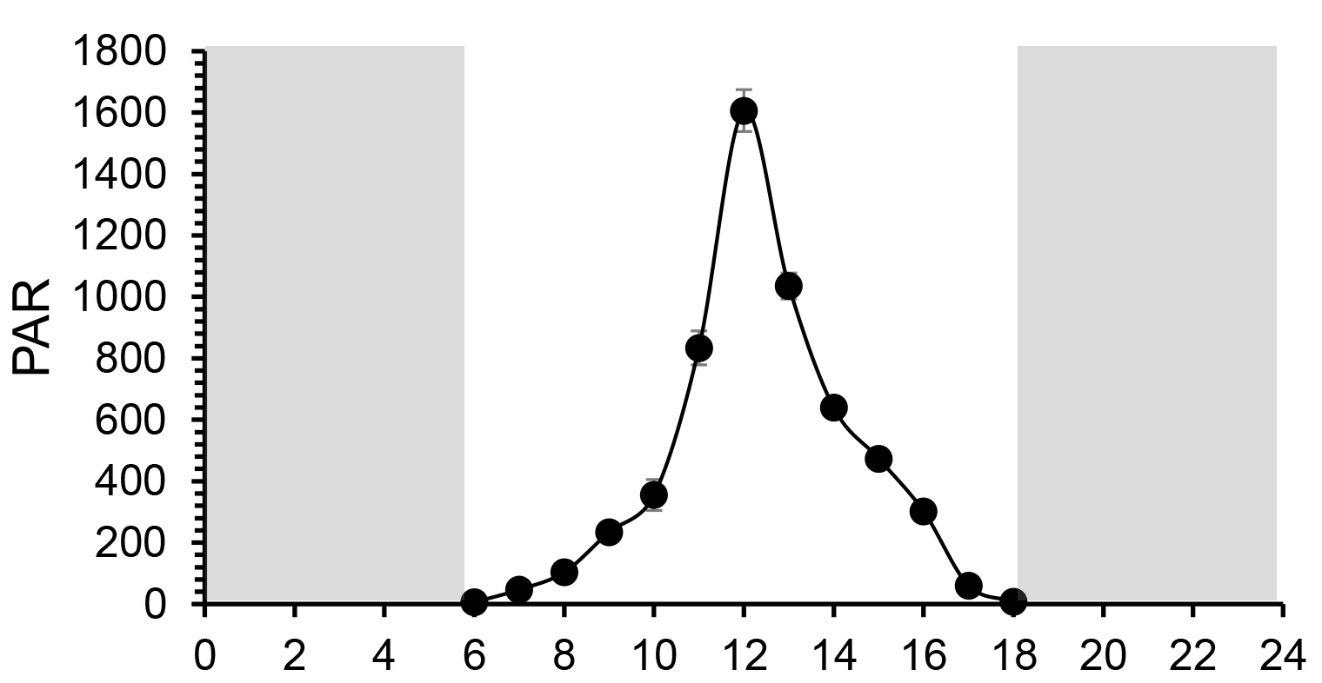


**S2 | Light intensity from morning to evening on 22^nd^ of June 2017 in field.**


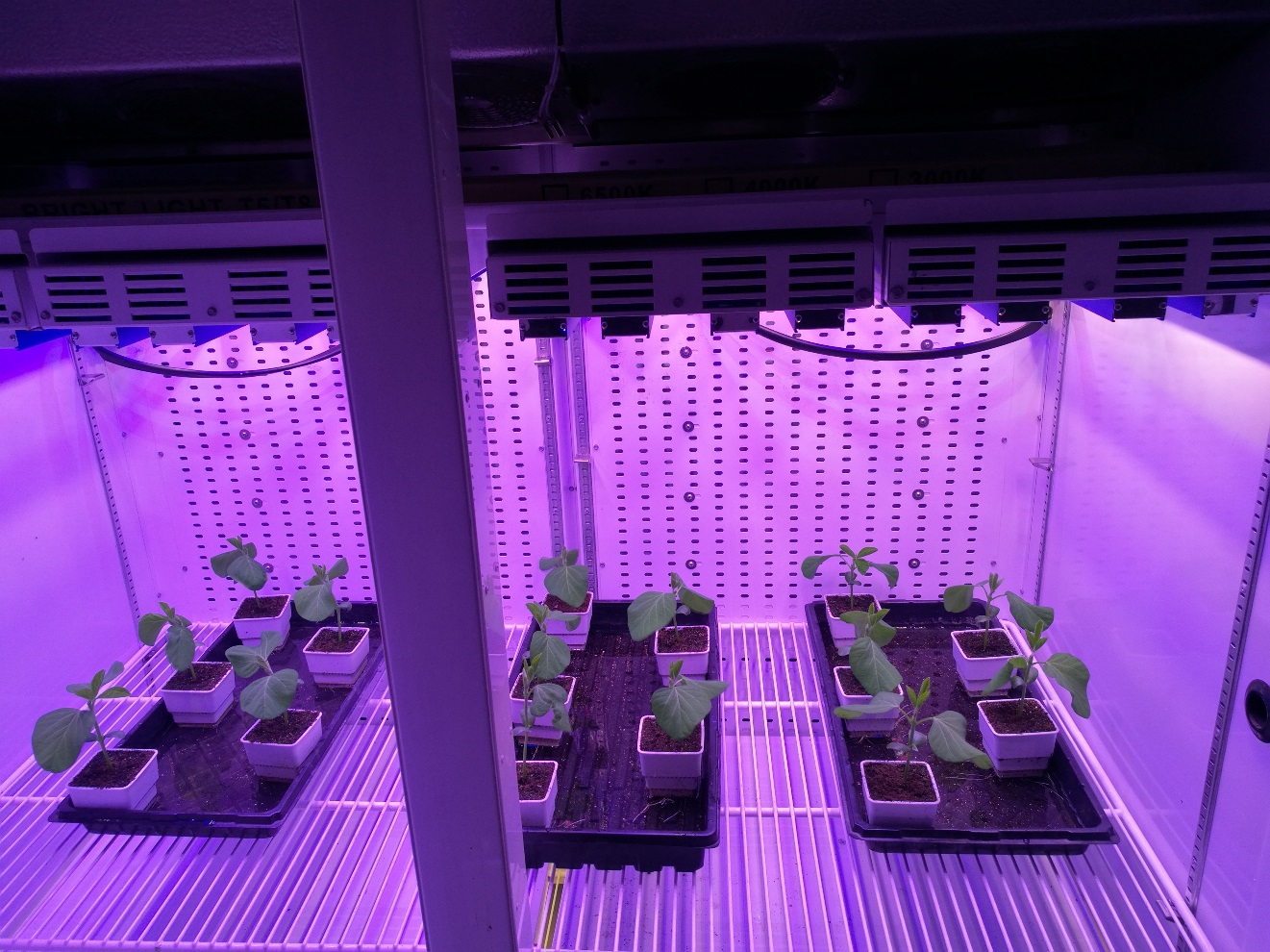


**S3 | Description of growth chamber.**

**TABLE S1 |**

**List of primers for characterizing soybean genes (**5' - 3'**) measured under different light treatments.**

| **Location** | **Gene** | **L primer** | **R primer** |
| --- | --- | --- | --- |
| *Glyma.13G161600* | *Gmsps1* | CTATACTATCCATCCCTCAATCCC | ATCAGCCCATCGCACTAAAG |
| *Glyma.17G109700* | *Gmsps2* | AGGTGACTCTGCTACTCTCCTATCT | TCCTTGCTCGTAGTTTACGCT |
| *Glyma.13G114000* | *Gmss1* | GGCATCCTGCAACACCAC | GAACAGCCAGAGCAACCC |
| *Glyma.02G304500* | *GmAGP1* | CAATTCAATTCCGCCTCGTTA | CTCATTTTTGTAGCCACCCATGT |
| *Glyma.19G104700* | *GmUGP1-1* | CGAATCTTCCACCGCAATG | GCGACGGCGGATTTGA |
| *Glyma.02G241100* | *GmUGP1-2* | CCACCGCCACCGAGAAG | CTCACTCTCACTGATTTCGTTCAATC |
| *Glyma.13G152500* | *GmUGP2* | GGCAGGGACGGGTGGTA | TGTGATAATAGCACATCAAGCTTTCC |
| *Glyma.04G235200* | *GmSSS1-1* | GCGGCGGTGCTACTTCA | CAAAATCGTCTGTTTCTCTGGCTAA |
| *Glyma.06G129400* | *GmSSS1-2* | AGACCAAACCTCTCACCACAAAG | GCAAGCGAAAGCCAACGA |
|  | *Gmactin11* | CCTCAACCCAAAGGTCAACAG | GACCAGCGAGATCCAAACGAA |

*Gmsps1* and *Gmsps2*, and *Gmss1* genes are involve in sucrose phosphate synthase and sucrose synthase, respectively. *GmAGP1 is* involve in ADP-glucose pyrophosphorylase. *GmUGP1-1*, *GmUGP1-2* and *GmUGP2-1*, *GmUGP2-2* are involve in UDP-glucose pyrophosphorylase, respectively. *GmSSS1-1*, *GmSSS1-2* are involve in soluble starch synthase.

**TABLE S2 |**

**Cross-sectional area of chloroplast organelles measured under different light treatments.**

| **Treatment** | **Chloroplast**  **(μm^2^)** | **Thylakoids**  **(μm^2^)** | **Starch grain**  **(μm^2^)** | **T:C** | **S:C** |
| --- | --- | --- | --- | --- | --- |
| L_100_ | 1300.31±18.67e | 684.30±13.78e | 55.84±1.05d | 0.53±0.010c | 0.04±0.002d |
| L_200_ | 1493.90±24.13d | 927.49±15.39d | 63.51±2.09d | 0.62±0.002b | 0.04±0.002d |
| L_300_ | 2119.89±25.94c | 1519.21±17.51c | 213.73±7.45c | 0.72±0.007a | 0.10±0.003c |
| L_400_ | 2451.84±24.83b | 1763.04±20.68a | 478.24±10.52b | 0.72±0.002a | 0.20±0.006b |
| L_500_ | 2648.93±30.62a | 1680.15±20.69b | 613.90±14.48a | 0.63±0.003b | 0.23±0.013a |

T:C and S:C represent the ratio between thylakoid and chloroplast, and starch grains and chloroplast, respectively. All the values are mean ± SD of three replicates and representative of three independent experiments. The different lowercase and same letters show a significant and non-significant at 5% level, according to LSD.
